# A Meta-Analysis of the Association Between Live Yeast Supplementation and Lactation Performance in Dairy Cows Under Heat Stress

**DOI:** 10.3390/ani16030428

**Published:** 2026-01-29

**Authors:** Babak Darabighane, Maria Giovanna Podda, Francesco Fancello, Alberto Stanislao Atzori

**Affiliations:** 1 Department of Agricultural Sciences, University of Sassari, 07100 Sassari, Italy; bdarabighane@uniss.it (B.D.); m.podda11@phd.uniss.it (M.G.P.); fancello@uniss.it (F.F.); 2 University School for Advanced Studies IUSS Pavia, 27100 Pavia, Italy; 3 Institute for Animal Production System in Mediterranean Environment, National Research Council (CNR—ISPAAM), 07100 Sassari, Italy

**Keywords:** milk losses, heat stress, live yeast, *Saccharomyces cerevisiae*, dairy cow

## Abstract

Heat stress is a major management challenge in dairy cows, leading to lower dry matter intake (DMI), reduced milk yield (MY), and changes in milk composition. A practical nutritional strategy is supplementing diets with live yeast (*Saccharomyces cerevisiae*) during hot periods. This meta-analysis evaluated the effects of live-yeast supplementation on DMI, MY, and milk components in heat-stressed dairy cows. Bibliographic searches for the period 2000 to 2025 were conducted. Based on the inclusion and exclusion criteria, 14 studies were selected. For each outcome, effect sizes were reported as the standardized mean difference (SMD) and the raw mean difference. Analyses were conducted using a random-effects model, and heterogeneity was assessed using the *Q* test and the *I*^2^ statistic. Based on the SMD, live-yeast supplementation of the diets of heat-stressed dairy cows increased both DMI (*p* = 0.003) and MY (*p* < 0.001). Milk fat percentage (MFP; *p* = 0.104) and milk lactose percentage (MLP; *p* = 0.530) were not significantly affected, whereas milk protein percentage (MPP) tended to increase (*p* = 0.078). In contrast, milk fat yield, milk protein yield (MPY), and milk lactose yield (MLY) all increased significantly (*p* ≤ 0.001). Heterogeneity was significant only for DMI, MPY, and MLY. Overall, based on the available evidence, supplementing heat-stressed dairy cows with live yeast can improve DMI and MY, and increase milk component yields, without materially altering component percentages.

## Simple Summary

Heat stress is a major challenge in dairy farming because it reduces feed intake, lowers milk yield, and can change milk composition, leading to economic losses and lower productivity. A practical nutritional strategy to support dairy cows during heat stress is live-yeast supplementation. The aim of this study was to evaluate whether live-yeast supplementation improves the performance of dairy cows exposed to heat stress. To address this question, we gathered results from previously published studies and analyzed them using a meta-analysis, a method that pools findings from multiple studies to estimate the overall effect. The results showed that supplementing the diet with live yeast in heat-stressed dairy cows increased dry matter intake and increased milk yield. In addition, the total yields of milk fat, protein, and lactose were higher. Overall, these findings suggest that live-yeast supplementation may be a practical nutritional option to help dairy cows maintain milk yield during heat stress.

## 1. Introduction

Global climate change is accompanied by an increase in mean ambient temperature and a rise in both the frequency and intensity of extreme weather events. The increased occurrence of heat waves may have substantial effects on the dairy industry in terms of productive performance and product quality [1,2]. Under these conditions, heat stress emerges as one of the most critical management challenges in the dairy sector. It occurs when concurrent increases in ambient temperature and humidity cause the temperature–humidity index (THI) to exceed a critical threshold. It is well established that when the THI reaches 72 or higher, dry matter intake (DMI) and milk yield (MY) decrease, and significant physiological changes are observed in high-producing cows [3]. Notably, THI levels and their impact depend on geographical location and animal type [4]. Heat stress further causes impaired rumen function, reduced rumination time, and decreased saliva production, ultimately reducing natural buffers and lowering rumen pH [5,6]. In addition, heat stress has been shown to alter the structure of the rumen microbiome, with lactate-producing species becoming more abundant and acetate-producing species less abundant [7], which can affect nutrient digestibility [6] and, ultimately, the rumen metabolism.

A proposed strategy to improve the performance of dairy cows under heat stress is the use of yeasts [8]. Previous studies have indicated that live yeast, by consuming ruminal oxygen and acting as a rich source of nutrients (such as B vitamins, organic acids, and amino acids), may support the growth of certain rumen bacteria, enhance the activity of cellulolytic and lactate-utilizing bacteria, and consequently contribute to the stabilization of ruminal pH [9,10,11]. Recent research has focused more on the use of live yeast compared to other yeast products, including yeast culture, in the context of dairy cows under heat stress. The reported results regarding the effect of live yeast on dairy cows under heat stress vary, and differences among studies may be related to factors such as the physiological state of the cow (e.g., stage of lactation), the dietary characteristics (e.g., forage-to-concentrate ratio), the dose of yeast, the method of administration, or the duration of the experiment. In an experiment where dairy cows were in mid-lactation and experienced a THI range of 76 to 87, the addition of live yeast (2.0 × 10^10^ CFU/g) at 10 and 20 g/day in the diet caused significant increases of 17.8% and 24.7% in DMI, respectively, and significant increases of 5.2% and 25.8% in MY compared to the control group [12]. In another experiment, where dairy cows were in early lactation and experienced a THI range of 74 to 83, the addition of live yeast (2.0 × 10^10^ CFU/g) at 0.5 and 1 g/day in the diet caused non-significant decreases of 2.9% and 2.4% in DMI, respectively, and non-significant increases of 1.6% and 3.8% in MY compared to the control group [13]. This lack of uniformity is also evident in the results of other studies on live-yeast supplementation in dairy cows under heat stress. Reports including data on rumen fermentation or nutrient digestibility could help explain the mechanism of action, but such information was unfortunately not provided in some studies. Therefore, considering the negative effects of heat stress on MY and the health of dairy cows, as well as the role of nutritional strategies in mitigating these effects, it is necessary to investigate the effect of live yeast under heat-stress conditions.

A comprehensive method to evaluate the results of different studies is particularly important to determine the overall effect of live yeast on the lactation performance of dairy cows under heat stress. Clarifying the effects of live yeast can contribute to increasing the productivity and profitability of dairy farms. Meta-analysis represents a statistical approach for combining treatment effects across studies using weighted estimates within fixed- or random-effects frameworks and assessing sources of heterogeneity [14]. This study aimed to perform a comprehensive meta-analysis of the effects of live-yeast supplementation on DMI, MY, and milk components in dairy cows under heat stress. To the best of our knowledge, this study represents the first work to provide a comprehensive quantitative synthesis of the effects of live-yeast supplementation in dairy cows exposed to heat stress, thereby contributing to an improved understanding of its relevance under these conditions.

## 2. Materials and Methods

### 2.1. Identification of Relevant Studies

Studies published in English between 2000 and 2025 were systematically identified to evaluate the effects of live-yeast supplementation on DMI, MY, and milk components in dairy cows exposed to heat stress. The literature search used Web of Science, Google Scholar, and Scopus, and searches combined the following terms: (“yeast” OR “live yeast” OR “*Saccharomyces cerevisiae*”) AND (“heat stress” OR “THI” OR “temperature humidity index” OR “temperature–humidity index” OR summer OR “hot season”) AND (“dairy cow” OR “lactating cow” OR “dairy cattle” OR “cow”). Only articles published in peer-reviewed journals were considered. For Google Scholar, several thousand records were collected, and results were sorted in order of relevance and the screening of papers stopped after at least 20 records after the last relevant record was identified. To ensure thorough coverage, the reference lists of all eligible original and review articles were examined manually.

### 2.2. Inclusion and Exclusion Criteria

Figure S1 presents a PRISMA flow diagram [15] illustrating the data collection process for the meta-analysis. Following screening, 21 articles were reviewed for eligibility; 7 were excluded for the following reasons: yeast combined with organic minerals (*n* = 3), lack of DMI, MY, and milk component reports (*n* = 1), no report of yeast type or manufacturer (*n* = 1), and missing statistical indicators (*n* = 2). The remaining 14 articles met the inclusion criterion, which was the effect of live yeast (*Saccharomyces cerevisiae*) on DMI, MY, and milk components in heat-stressed dairy cows. 

### 2.3. Data Collection

The following variables were extracted from each study: DMI, MY, milk fat percentage (MFP), milk fat yield (MFY), milk protein percentage (MPP), milk protein yield (MPY), milk lactose percentage (MLP), and milk lactose yield (MLY). In some articles, values for MFY, MPY, and MLY were not reported; these values were calculated using MY and milk composition percentages, and standard errors were estimated using the error propagation technique. The DMI values shown in figures were extracted with WebPlotDigitizer (https://automeris.io/). Data on the number of animals in control and treatment groups were extracted, together with the reported standard deviation (SD) or standard error (SE). When SD was not reported, it was derived by multiplying the reported SE of the mean by the square root of the corresponding sample size. Additional data covered dairy cow characteristics (breed, body weight, days in milk (DIM), and parity); feed characteristics (forage type, forage-to-concentrate ratio, neutral detergent fiber (NDF), crude protein (CP), and net energy for lactation (NEL)); live-yeast characteristics (dose, delivery method, and manufacturer when reported); and information on the start and end of heat-stress exposure, together with THI. In several studies, the mean and maximum ambient temperature and relative humidity over the experimental period were either not reported or not provided in tables; in others, they appeared only as figures, which did not allow for reliable numerical extraction. In some cases, the THI formula was also not specified. As a result, recalculating and harmonizing THI with a single reference formula across studies was not possible. Because rumen fermentation variables and nutrient digestibility outcomes were inconsistently reported in a subset of studies and the number of treatment–control comparisons available for meta-analysis was small (fewer than 10 independent contrasts), we did not analyze these outcomes to avoid bias and to maintain statistical validity [16]. The same decision applied to respiration rate and rectal temperature: in several reports, these measures were either missing or taken at unspecified times of day (e.g., morning vs. afternoon), which further reduced the number of usable contrasts; therefore, these variables were also excluded from the analysis. All extracted data were entered into Excel (Microsoft Corporation, Redmond, WA, USA). Before any analysis, data extractors were trained and pilot-tested on a standardized extraction form; outcome data and other items requiring judgment were then extracted independently by two reviewers, with discrepancies resolved by discussion to minimize errors and bias [17].

### 2.4. Statistical Analysis

Statistical analyses were carried out using Comprehensive Meta-Analysis (CMA), version 4 (Biostat, Addison, TX, USA). Effect sizes were computed as the standardized mean difference (SMD) and the raw mean difference (RMD), each with a 95% confidence interval (CI). Standardized mean difference is the difference between the treatment and control means, scaled by the SD of the two groups [18]. It was calculated using the following formula:

$$SMD=\frac{{\bar{x}}_{e}-{\bar{x}}_{c}}{S_{p}}$$

where ${\bar{x}}_{e}$ and ${\bar{x}}_{c}$ are the mean values for treatment and control groups, respectively, and S_p_ is the pooled SD [19]. The raw mean difference reflects the difference between treatment and control groups and expresses the effect size in the original units of measurement. A random-effects model was used for the meta-analysis, assuming a distribution of true effects and, therefore, heterogeneity across studies [18]. Statistical significance for both SMD and RMD estimates was defined at a *p*-value of 0.05 or less. Forest plots were generated for DMI and MY, with effect sizes expressed as SMD and 95% CI under the random-effects model.

We computed prediction intervals (PIs; 95%) on the SMD scale using a random-effects model. Prediction intervals quantify the range within which the true effect in a new, comparable study is expected to lie. In contrast to confidence intervals, which reflect the precision of the pooled mean, PIs summarize between-study dispersion and the expected range of true effects on the SMD scale. When the between-study variance was estimated to be negligible (τ^2^ = 0), PIs were not reported [18].

Statistical heterogeneity reflects differences in the underlying effects across studies [14]. Such heterogeneity may arise from differences in herd characteristics, animal health status, study design, or random statistical variation [19]. We assessed heterogeneity using Cochran’s *Q* test and the *I*^2^ statistic [18]. Between-study variance was examined with *Q*; given the relatively low power of *Q* when only a few studies are included, we used a significance level of 0.10 [19]. While *Q* helps detect heterogeneity, *I*^2^ was used to quantify its magnitude as a percentage [19].

$$I^{2}\left(\%\right)=\frac{Q-\left(k-1\right)}{Q}\times 100$$

where *Q* denotes the chi-square statistic for heterogeneity and k represents the number of trials.

The *I*^2^ index was interpreted using established thresholds. Values of 75–100% indicate considerable heterogeneity, 50–90% may represent substantial heterogeneity, 30–60% may represent moderate heterogeneity, and 0–40% may be unimportant. Negative *I*^2^ values were set to zero [20].

Meta-regression analyses were conducted to investigate potential sources of heterogeneity, with the study-specific SMDs used as the dependent variable and the corresponding SE serving as the measure of variance. For outcomes with evidence of heterogeneity based on Cochran’s *Q* test (*p* < 0.10), meta-regression analyses were performed to examine potential contributing factors. Models were fit under a random-effects framework with the between-study variance (τ^2^) estimated by restricted maximum likelihood (REML). Inference used two-sided *p*-values with 95% confidence intervals and the Knapp–Hartung small-sample adjustment [18]. Covariates considered in the heterogeneity analyses included DIM, the proportion of forage in the diet (FOR), and the treatment–control difference in milk yield.

Because meta-analytic estimates may remain biased when the available studies represent a selective subset of the evidence, publication bias was assessed by examining funnel-plot asymmetry with Egger’s regression test [21]. When evidence of asymmetry was observed (*p* < 0.10), the trim-and-fill procedure was used to estimate the potential number of missing studies and to adjust the pooled effect estimate accordingly [22]. Funnel plots were examined to visually assess asymmetry, under the assumption that, in the absence of publication bias, effect estimates are expected to be symmetrically distributed around the underlying true effect.

## 3. Results

### 3.1. Database Summary

A summary of the 14 studies included in this meta-analysis is presented in Table 1. In most studies, the cattle breed was Holstein; in the studies by Er and Cengiz [23] and Lees et al. [24], the Holstein-Friesian breed was used, and in the study by Moallem et al. [25], the Israeli-Holstein breed was used. In five studies, the lactation stage of the cows was early-lactation [13,26,27,28,29], in eight studies it was mid-lactation [12,23,24,25,30,31,32,33], and in one study it was late-lactation [34]. In some studies, the initial body weight of the cows was not reported [12,24,29,31,34], and among the studies that did report it, the lowest weight was 587 kg [25] and the highest was 743 kg [26]. Parity status was also not reported in many studies; however, in those that did provide this information, the average parity was 2.98. The forage base in the dairy cow diet in most studies consisted of corn silage and alfalfa hay. The average forage-to-concentrate ratio was 45.5:54.5, and the mean values for NDF, CP, and NEL were 32.19%, 16.6%, and 1.62 Mcal/kg DM, respectively. The delivery method of live yeast varied across studies, being used as an in-feed additive in the mixed ration in Lees et al. [24], mixed with water in Er and Cengiz [23], and used as a top-dressing in other studies. Details on the yeast strain, product name, manufacturer, and administered dose are provided in Table S1. In most studies, based on product specifications and manufacturer documentation, the yeast used was *Saccharomyces cerevisiae* CNCM I-1077 [13,23,24,27,28]. The administered doses and, consequently, the CFU delivered varied across studies. In studies reporting CFU per gram, the minimum and maximum values were 2.5 × 10^8^ and 2.0 × 10^10^ CFU/g, respectively. In one study, cows were tested in climatic chambers simulating heat environments [30], while in other studies, cows were tested under seasonal conditions. Table 1 summarizes the start and end times of heat-stress exposure by country.

### 3.2. Dry Matter Intake and Milk Yield

**Figure 1:**
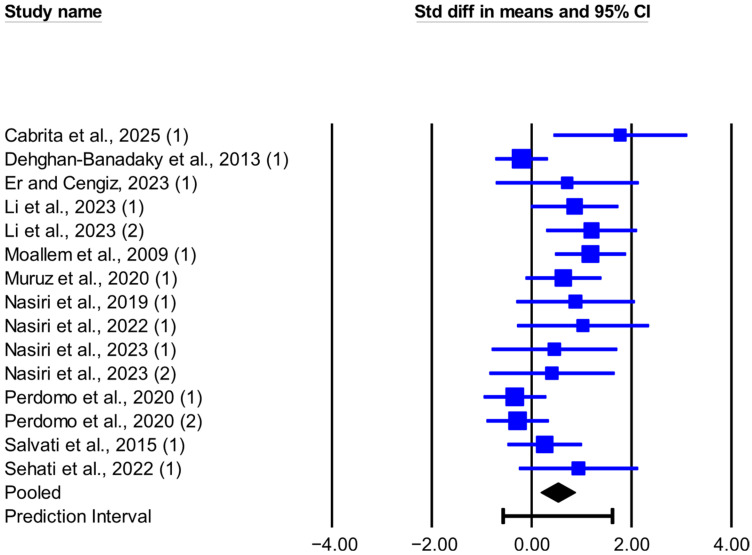
Forest plot of the effect of live yeast supplementation on DMI in dairy cows under heat stress, expressed as standardized mean difference (Std diff in means). Squares are proportional to study weights; horizontal blue lines are 95% CIs. The diamond indicates the random-effects pooled SMD, with the horizontal black bar showing the overall 95% prediction interval. Numbers in parentheses following each study citation indicate the comparison number within that study. The following studies are referenced in the plot: [12,13,23,25,26,27,28,29,30,31,33,34].

**Figure 2:**
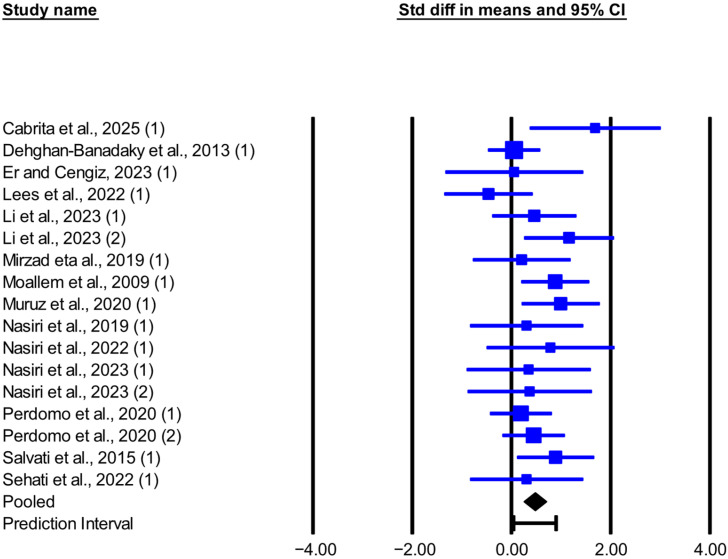
Forest plot of the effect of live yeast supplementation on MY in dairy cows under heat stress, expressed as standardized mean difference (Std diff in means). Squares are proportional to study weights; horizontal blue lines are 95% CIs. The diamond indicates the random-effects pooled SMD, with the horizontal black bar showing the overall 95% prediction interval. Numbers in parentheses following each study citation indicate the comparison number within that study. The following studies are referenced in the plot: [12,13,23,24,25,26,27,28,29,30,31,32,33,34].

The meta-analysis results, heterogeneity, and publication bias for the effect of live-yeast supplementation on DMI and MY in dairy cows under heat stress are presented in Table 2. Live-yeast supplementation significantly increased DMI (SMD = 0.526; *p* = 0.003; Figure 1), with a 95% prediction interval of −0.570 to 1.621 SMD. Based on the RMD effect size, DMI increased by 0.304 kg/d. Live-yeast supplementation also significantly increased MY (SMD = 0.473; *p* < 0.001; Figure 2), with a 95% prediction interval of 0.045 to 0.900 SMD. According to the RMD effect size, MY increased by 0.749 kg/d.

**Figure 3:**
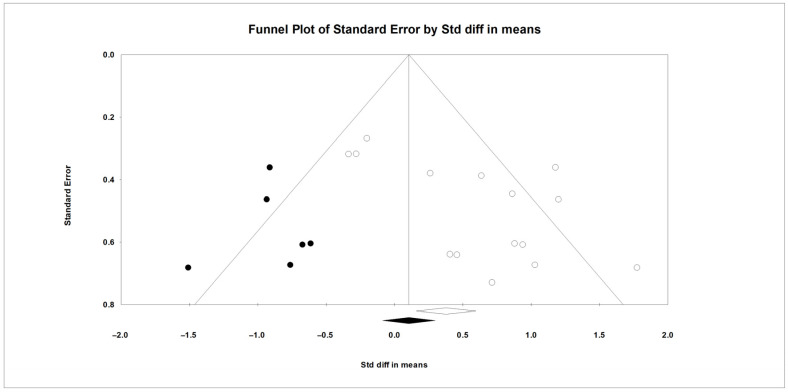
The funnel plot shows the standardized mean differences (Std diff in means) for studies on DMI, with empty circles representing the included studies. Solid dots indicate potentially missing studies imputed using the trim-and-fill method. The open diamond represents the mean and CI based on the existing studies, while the solid diamond shows the mean and CI if the theoretically imputed studies were included in the meta-analysis.

In this meta-analysis, between-study heterogeneity was significant for DMI but not for MY (*Q* and *I*^2^, Table 2). Meta-regression analyses showed that DIM and FOR were not associated with the observed heterogeneity in DMI (Table S2). Results from Egger’s test suggested the presence of publication bias for DMI (*p* < 0.1), and the trim-and-fill method suggested six missing observations on the left side of the funnel plot (Figure 3); no evidence of publication bias was observed for MY (*p* > 0.1).

### 3.3. Milk Composition

The meta-analysis results, heterogeneity, and publication bias for milk composition under heat stress are reported in Table 3. MFP (SMD = 0.220; *p* = 0.104) and MLP (SMD = 0.086; *p* = 0.530) did not increase significantly, whereas MPP tended to increase (SMD = 0.208; *p* = 0.078). The 95% prediction intervals were −0.473 to 0.912 SMD for MFP, −0.557 to 0.730 SMD for MLP, and −0.196 to 0.611 SMD for MPP. No significant between-study heterogeneity was observed for percentage-based milk composition (*Q* and *I^2^*, Table 3), and no publication bias was observed (*p* > 0.1). MFY, MPY, and MLY increased significantly (*p* ≤ 0.001). The 95% prediction interval was not reported for MFY because the between-study variance was estimated to be approximately zero; it was −0.215 to 1.641 SMD for MPY and −0.597 to 1.865 SMD for MLY. Heterogeneity was insignificant for MFY, but significant for MPY and MLY (*Q* and *I*^2^, Table 3). The meta-regression analysis indicated that differences in MY contributed to the heterogeneity observed for MPY and MLY (Table S2), and Egger’s test suggested no evidence of publication bias (*p* > 0.1).

## 4. Discussion

It is well documented that signs of heat stress in dairy cows occur when the THI exceeds 72 [35,36] and that heat stress can have detrimental effects, including increased respiratory rate, elevated body temperature, decreased DMI, altered rumen fermentation patterns, reduced milk production, and altered milk composition [37,38]. To mitigate these consequences, various nutritional approaches, including dietary microbial additives, have been proposed [8,37]. In recent years, the use of yeast products, especially live *Saccharomyces cerevisiae*, as a dietary supplement for dairy cows under heat stress has been widely investigated. Based on this evidence, this meta-analysis quantitatively assesses the effect of live yeast on performance indicators for dairy cows experiencing heat stress.

The results of this meta-analysis show that adding live yeast to the diet of dairy cows under heat stress significantly increased DMI by about 0.3 kg/day. This rise in DMI aligns with most studies included here. In Li et al. [12], with THI between 76 and 87, supplementing 10 or 20 g/day of live yeast (2.0 × 10^10^ CFU/g) increased DMI by 17.8% and 24.7% versus the control. This increase was accompanied by a lower respiratory rate and rectal temperature in cows receiving yeast. By contrast, in Perdomo et al. [13], with a THI between 74 and 83, supplementation with 0.5 or 1 g/day of live yeast (2.0 × 10^10^ CFU/g) produced non-significant decreases in DMI of 2.9% and 2.4% versus the control. The respiratory rate increased and the rectal temperature did not change. In Er and Cengiz [23], where the daily average THI was above 76, 1 g/day of live yeast (10 × 10^9^ CFU/g) caused no significant changes in DMI, respiratory rate, or rectal temperature relative to the control. The evidence suggests that the effects of live yeast on respiratory rate and thermoregulation, and thus on DMI, may depend on the yeast strain and dose, and further evidence is needed to draw firm conclusions. In this meta-analysis, the 95% prediction interval (based on SMD) for DMI indicated that, in comparable future herds, the response could range from no effect or slightly negative to a significant increase. However, inconsistencies across studies may reflect factors such as physiological state, stage of lactation, type of feed, yeast dose, severity of heat stress, and the length of the adaptation period to supplementation. In this context, Chen et al. [39] reported in a meta-analysis of heat stress in dairy cows that DMI showed significant heterogeneity, which could relate to both the duration and severity of heat stress at different stages of lactation. The meta-regression analysis for DMI showed that DIM and FOR did not contribute to the observed heterogeneity. Therefore, its origin is likely to be clinical variability (animal characteristics, intervention type, or outcome measurement method) or methodological variability among studies. Identifying these sources will help target management strategies more accurately and improve the design of future studies [19]. Egger’s test indicated funnel asymmetry, which is consistent with small-study effects [18]. Furthermore, results from the trim-and-fill analysis pointed to six potentially missing studies on the left side of the funnel plot. After imputation, the adjusted SMD was smaller than the original estimate (Figure 3), a pattern consistent with the overestimation of the effect size in the original analysis. Given the significant heterogeneity, between-study differences can themselves contribute to asymmetry.

This meta-analysis showed that supplementing live yeast in the diets of heat-stressed dairy cows significantly increases MY by about 0.75 kg/day. This result is consistent with most included studies except for Dehghan-Banadaky et al. [31], Er and Cengiz [23], and Lees et al. [24]. Because the 95% prediction interval is above zero, the true effect on MY is expected to be positive in most comparable future herds, although its magnitude likely ranges from small to moderate. Across studies, the percentage increase in MY for live-yeast groups versus controls varied. In Li et al. [12], cows receiving 20 g/day of live yeast (2.0 × 10^10^ CFU/g) at 187 DIM showed the largest increase, 25.8%, while cows receiving 10 g/day showed a 5.2% increase. The improvement in MY in response to live yeast appears to reflect the interplay of several physiological and microbial processes, including enhanced rumen fermentation, greater pH stability, and beneficial shifts in the microbial community. The precise mechanisms and the relative contribution of each factor still require further clarification. Abdelli et al. [40], in a meta-analysis of yeast products (including live yeast and yeast culture) in dairy cows, argued that the observed increase in MY in cows receiving yeast products was likely a consequence of improved rumen function and feed efficiency rather than an increase in DMI. On the other hand, Perdomo et al. [13] reported that, in heat-stressed cows, live yeast increased the rumen pH and reduced the proportion of cows with a rumen pH below 5.80, the proportion with measurable lactate, and the proportion with lactate greater than 1 mmol/L in the rumen fluid. This suggests that adding live yeast to the diet may promote ruminal oxygen scavenging, support the activity of cellulolytic and lactate-utilizing bacteria, and contribute to ruminal pH stabilization [9,41], thereby benefiting intake regulation, feeding behavior, and NDF digestion. According to the reported evidence, improving nutrient digestibility and increasing dietary digestible energy can support higher MY [12,13,42]. Although this meta-analysis did not evaluate apparent nutrient digestibility due to limited data and non-reporting in some articles, studies that reported these parameters found increased nutrient digestibility and subsequent MY in heat-stressed cows receiving live yeast [13,28]. In contrast, in two studies where yeast supplementation did not alter nutrient digestibility, MY was not significantly affected [23,31]. This pattern supports a relationship between improved nutrient digestibility and the production response, although the body of evidence is limited and requires further reporting. Another possible mechanism is that cows rely more on glucose as an energy source under heat stress, and increased glucose availability to the mammary gland in response to yeast supplementation may enhance lactose synthesis and thus improve MY [34]. In Salvati et al. [34], supplementation with 10 g/day of yeast (equivalent to 25 × 10^10^ CFU of live cells and 5 × 10^10^ CFU of dead cells) increased plasma glucose concentrations in the yeast group compared with the control, and MY increased by approximately 1.3 kg/day.

The findings of this meta-analysis show that supplementation with live yeast did not change MFP in dairy cows under heat stress. This finding agrees with most of the included studies except for Sehati et al. [29], Nasiri et al. [26], and Dehghan-Banadaky et al. [31]. In Dehghan-Banadaky et al. [31], where THI ranged from 76.8 to 79.34, supplementation with 4 g/day of live yeast (15 × 10^9^ CFU/g) increased MFP by 5.3% compared with the control. The authors attributed this increase to shifts in rumen fermentation in response to live yeast and to the greater availability of lipogenic precursors. For MPP, this meta-analysis showed a trend toward an increase, although most studies reported a non-significant effect. Perdomo et al. [13] reported a significant increase, and Cabrita et al. [30] and Li et al. [12] reported that MPP tended to increase. For MLP, this meta-analysis showed no effect of live-yeast supplementation on heat-stressed cows. Consistent with this, most studies reported non-significant effects, although Cabrita et al. [30] and Dehghan-Banadaky et al. [31] observed a tendency to decrease. In this meta-analysis, milk component yields increased significantly. Based on the RMD effect size, the estimates for MFY, MPY, and MLY were 0.033, 0.037, and 0.059, respectively. Heterogeneity was significant for MPY and MLY, and meta-regression analysis indicated that differences in MY between the live yeast and control groups were a source of this heterogeneity. For example, in Li et al. [12], cows receiving 20 g/day of live yeast (2.0 × 10^10^ CFU/g) showed a 25.8% increase in MY compared with the control group. This large effect size increased the analytical weight of the study and added to the heterogeneity in MPY and MLY. These findings suggest that the improvement in component yield largely reflects an increased milk volume rather than independent changes in the biological pathways of milk composition. The 95% prediction intervals illustrate the dispersion of effects in comparable future herds. For MPY, the response ranges from slightly negative or near zero to large increases, whereas for MLY, the range is broader, spanning from negative to very large increases.

Several limitations should be taken into account when interpreting the findings. First, although all included studies stated in the title or Materials and Methods that cows were exposed to heat stress and specified the start and finish seasons or months, several studies did not fully report temperature and humidity data or the exact THI calculation formula. This information is fundamental in heat-stress research, and its absence prevented the use of THI (or its components) as covariates in meta-regression analysis and limited precise between-study comparisons. Second, the number of reported data points on rumen fermentation and apparent nutrient digestibility was small. Statistical power was therefore insufficient to analyze these outcomes specifically, and we could not reconstruct the biological pathways by which live yeast may act in heat-stressed cows. Third, insufficient reporting of product CFU data created a gap that limited dose–response modeling and testing dose as a source of heterogeneity. Fourth, incomplete reporting of physiological status (stage of lactation/DIM, body condition score) and detailed diet chemical composition prevented us from including these variables as covariates in the meta-regression analyses. Nevertheless, this study allowed for the quantification of the most relevant production-level responses of heat-stressed dairy cows to live-yeast supplementation, including feed intake, MY, and milk component responses. To strengthen the evidence base and enable more precise causal inference, future studies should adopt a uniform reporting framework. This framework should include standardized recording of temperature, humidity, and a documented THI formula; systematic reporting of product CFU/g (measured in the laboratory during the experiment), and CFU/day; regular measurement of rumen-fermentation indices and nutrient digestibility; and complete documentation of animal physiological status and diet chemical composition. Implementing these practices will support a clearer understanding and more actionable guidance on the effects of live yeast on the performance of heat-stressed dairy cows.

## 5. Conclusions

This meta-analysis shows that live-yeast supplementation in heat-stressed dairy cows increases DMI by roughly 0.3 kg/d and MY by about 0.75 kg/d, resulting in greater yields of milk fat, protein, and lactose without significant changes in their percentages. Therefore, live-yeast supplementation can be considered a practical nutritional strategy to mitigate the consequences of heat stress on the lactation performance of dairy cows. Due to the limitations of the available evidence, future studies are needed to deepen understanding and consolidate the results. To clarify the mechanisms by which live yeast acts in heat-stressed dairy cows, it is recommended to focus on feeding behaviors, nutrient digestibility, rumen fermentation parameters, the rumen microbiota, and blood biomarkers under heat-stress conditions that should be measured and reported.

## Figures and Tables

**Table 1:** Summary of papers used for the meta-analysis.

References	NC ^a^	Country	Breed	Lactation Stage ^b^	Body Weight	Forage Type	Start and Finish Exposed to Heat Stress
Cabrita et al., 2025 [30]	1	Portugal	Holstein	mid	600	Maize silage and Ryegrass hay	climatic chambers
Dehghan-Banadaky et al., 2013 [31]	1	Iran	Holstein	mid	NR ^c^	Corn silage and Alfalfa hay	July to August
Er and Cengiz, 2023 [23]	1	Turkey	Holstein-Friesian	mid	609	Corn silage and Alfalfa hay	June to September
Lees et al., 2022 [24]	1	Australia	Holstein-Friesian	mid	NR	Barley silage, Corn silage and Barley hay	December to January
Li et al., 2023 [12]	2	China	Holstein	mid	NR	Barley silage, Leymus chinensis hay and Alfalfa hay	July to August
Mirzad et al., 2019 [32]	1	Japan	Holstein	mid	715	Alfalfa hay, Oat hay and Wrapped bale silage of Italian ryegrass	July to September
Moallem et al., 2009 [25]	1	Israel	Israeli-Holstein	mid	587.15	Wheat silage, Vetch hay and Oat hay	July to October
Muruz et al., 2020 [33]	1	Turkey	Holstein	mid	634	Corn silage and Alfalfa hay	June to August
Nasiri et al., 2019 [26]	1	Iran	Holstein	early	743	Corn silage and Alfalfa hay	July to October
Nasiri et al., 2022 [27]	1	Iran	Holstein	early	670	Corn silage and Alfalfa hay	July to September
Nasiri et al., 2023 [28]	2	Iran	Holstein	early	650	Corn silage and Alfalfa hay	July to September
Perdomo et al., 2020 [13]	2	USA	Holstein	early	610	Corn silage and Alfalfa hay	May to September
Salvati et al., 2015 [34]	1	Brazil	Holstein	late	NR	Corn silage and Tifton silage	January to April
Sehati et al., 2022 [29]	1	Iran	Holstein	early	NR	Corn silage and Alfalfa hay	June to August

^a–c^ NC, number of comparisons (number of comparisons between the mean treatment group and the mean control group); according to DIM, the lactation stage was categorized into three classes, early lactation (DIM ≤ 100), mid lactation (101 ≤ DIM ≤ 200), and late lactation (DIM > 200); NR, Not reported.

**Table 2:** Effect size, heterogeneity, and publication bias for the effects of live yeast supplementation on dry matter intake and milk yield in dairy cow under heat-stress conditions.

Outcome ^a^	NC ^b^	SMD ^c^ (95% CI ^d^)		95% PIs ^e^	Heterogeneity			Publication Bias	RMD ^f^ (95% CI)
		Random Effect	*p*-Value		*Q*	*p*-Value	*I* ^2^		
DMI	15	0.526 (0.184, 0.867)	0.003	−0.570, 1.621	30.667	0.006	54.349	0.008	0.304 (0.063, 0.545)
MY	17	0.473 (0.248, 0.697)	<0.001	0.045, 0.900	18.263	0.309	12.393	0.425	0.749 (0.295, 1.204)

^a–f^ DMI, dry matter intake; MY, milk yield; NC, count of comparisons (number of comparisons between the mean treatment group and the mean control group); SMD, standardized mean difference; CI, confidence interval; PIs, prediction intervals; RMD, raw mean difference.

**Table 3:** Effect size, heterogeneity, and publication bias for the effects of live yeast supplementation on milk composition in dairy cow under heat-stress conditions.

Outcome ^a^	NC ^b^	SMD ^c^ (95% CI ^d^)		95% PIs ^e^	Heterogeneity			Publication Bias	RMD ^f^ (95% CI)
		Random Effect	*p*-Value		*Q*	*p*-Value	*I* ^2^		
MFP	16	0.220 (−0.045, 0.485)	0.104	−0.473, 0.912	21.841	0.112	31.321	0.429	0.050 (−0.003, 0.103)
MFY	16	0.548 (0.336, 0.759)	<0.001	-	11.703	0.701	0	0.965	0.033 (0.018, 0.047)
MPP	14	0.208 (−0.203, 0.439)	0.078	−0.196, 0.611	14.531	0.338	10.534	0.336	0.020 (0.002, 0.038)
MPY	14	0.713 (0.397, 1.029)	<0.001	−0.215, 1.641	23.979	0.031	45.785	0.209	0.037 (0.020, 0.053)
MLP	13	0.086 (−0.184, 0.357)	0.530	−0.557, 0.730	16.728	0.160	28.265	0.120	0.004 (−0.024, 0.032)
MLY	13	0.634 (0.253, 1.015)	0.001	−0.597, 1.865	30.484	0.002	60.635	0.101	0.059 (0.022, 0.096)

^a–f^ MFP, milk fat percentage; MFY, milk fat yield; MPP, milk protein percentage; MPY, milk protein yield; MLP, milk lactose percentage; MLY, milk lactose yield; NC, count of comparisons (number of comparisons between the mean treatment group and the mean control group); SMD, standardized mean difference; CI, confidence interval; PIs, prediction intervals; RMD, raw mean difference.

## Data Availability

All data analyzed in this study were obtained from previously published sources, which are fully listed in the reference section. No new experimental data were produced for this study. All information necessary to support the findings of this meta-analysis is available within the cited literature.

## Supplementary Materials

The following supporting information can be downloaded at: https://www.mdpi.com/article/10.3390/ani16030428/s1, Figure S1: The PRISMA flow diagram of the systematic review from initial search and screening to final selection of publications to be included in the meta-analysis; Table S1: Details of yeast strain, product name, manufacturer, and administered dose in included studies; Table S2: Summary of meta-regression analysis.
