# Supplementary material for: A Meta-Analysis of the Association Between Live Yeast Supplementation and Lactation Performance in Dairy Cows Under Heat Stress
Source: Animals (Basel). 2026 Jan 29;16(3):428. doi: 10.3390/ani16030428 (PMC12897254; doi:10.3390/ani16030428)
Supplement: Supplementary file 1 [file animals-16-00428-s001.zip › Supplementary, Jan 27.pdf]

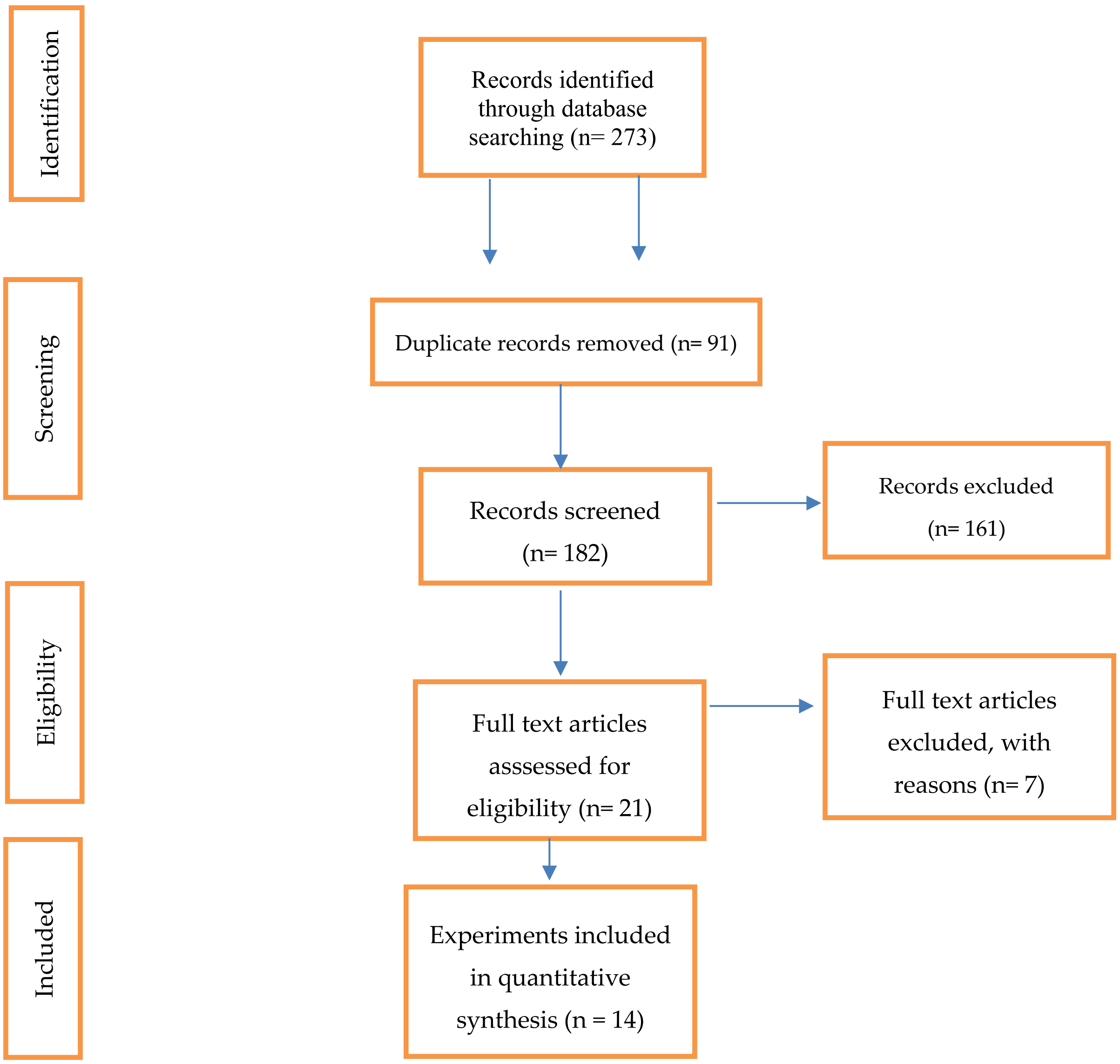

Figure S1. The PRISMA flow diagram of the systematic review from initial search and screening to final selection of publications to be included in the meta-analysis.

Table S1. Details of yeast strain, product name, manufacturer, and administered dose in included studies.

References	Species, Strain	Product name	Manufacturer	Dose	Colony-forming units
Cabrita et al., 2025	*S. cerevisiae*, 1026	Yea-Sacc	Alltech	0.5 g/kg TMR DM	2.5×10^8 CFU/g
Dehghan-Banadaky et al., 2013	*S. cerevisiae*, MUCL 39885	Probio-Sacc	Biochem	4 g/day/head	15×10^9 CFU/g
Er and Cengiz, 2023	*S. cerevisiae*, CNCM I-1077	Levucell SC 10 ME Titan	Lallemand Animal Nutrition	1 g/day/head	10×10^9 CFU/g
Lees et al., 2022	*S. cerevisiae*, CNCM I-1077	Levucell	Lallemand Animal Nutrition	50 g/day/head	10^{10} CFU/day
Li et al., 2023	*S. cerevisiae*, Y03-0	-	Shandong Shengqi Biological	10, 20 g/day/head	2.0×10^{10} CFU/g
Mirzad et al., 2019	*S. cerevisiae*, -	ActiSaf	Phileo Lesaffre Animal Care	10 g/day/head	-
Moallem et al., 2009	*S. cerevisiae*, SC47	Biosaf	Lesaffre Feed Additives	1 g per 4 kg of DM consumed	10^{10} CFU/g
Muruz et al., 2020	*S. cerevisiae*, -	-	BRT Feed Additives	5 g/day/head	10^8 CFU/day
Nasiri et al., 2019	*S. cerevisiae*, MUCL 39885	Probio-Sacc	Biochem	4 g/day/head	15×10^9 CFU/g
Nasiri et al., 2022	*S. cerevisiae*, CNCM I-1077	Levucell SC 20	Lallemand Animal Nutrition	6 g/day/head	10×10^9 CFU/g
Nasiri et al., 2023	*S. cerevisiae*, CNCM I-1077	Levucell SC 20	Lallemand Animal Nutrition	0.5, 1 g/day/head	10×10^9 CFU/g
Perdomo et al., 2020	*S. cerevisiae*, CNCM I-1077	Levucell SC 20	Lallemand Animal Nutrition	0.5, 1 g/day/head	1.42×10^{10} and 3.76×10^{10} CFU/day
Salvati et al., 2015	*S. cerevisiae*, NCYC 996	Procreatin-7	Lesaffre Feed Additives	10 g/day/head	25×10^{10} CFU/day
Sehati et al., 2022	*S. cerevisiae*, MUCL 39885	Probio-Sacc	Biochem	4 g/day/head	15×10^9 CFU/g

Table S2. Summary of meta-regression analysis.

**Outcomes**[a]	**Covariate**[b]	**Slope**	***p*-value**	**Intercept**	***p*-value**
DMI	DIM	0.002	0.464	0.339	0.283
	FOR	-0.0003	0.988	0.545	0.531
MPY	MYD	0.339	0.037	0.283	0.224
MLY	MYD	0.561	0.001	-0.093	0.635

[a-b] DMI, dry matter intake; MPY, milk protein yield; MLY, milk lactose yield; DIM, days in milk; FOR, dietary forage proportion; MYD, milk yield difference (treatment - control).
